# A Diagnostic Accuracy Study of Xpert®MTB/RIF in HIV-Positive Patients with High Clinical Suspicion of Pulmonary Tuberculosis in Lima, Peru

**DOI:** 10.1371/journal.pone.0044626

**Published:** 2012-09-07

**Authors:** Gabriela Carriquiry, Larissa Otero, Elsa González-Lagos, Carlos Zamudio, Eduardo Sánchez, Pamela Nabeta, Miguel Campos, Juan Echevarría, Carlos Seas, Eduardo Gotuzzo

**Affiliations:** 1 Facultad de Medicina Alberto Hurtado, Universidad Peruana Cayetano Heredia, Lima, Peru; 2 Instituto de Medicina Tropical Alexander von Humboldt, Universidad Peruana Cayetano Heredia, Lima, Peru; 3 Servicio de Enfermedades Infecciosas y Tropicales, Hospital Nacional Hipólito Unánue, Lima, Peru; 4 Foundation for Innovative New Diagnostics, Geneva, Switzerland; 5 Departamento de Enfermedades Infecciosas y Tropicales, Hospital Nacional Cayetano Heredia, Lima, Peru; San Francisco General Hospital, University of California San Francisco, United States of America

## Abstract

**Background:**

Diagnosis of pulmonary tuberculosis (TB) among human immunodeficiency virus (HIV) patients remains complex and demands easy to perform and accurate tests. Xpert®MTB/RIF (MTB/RIF) is a molecular TB diagnostic test which is rapid and convenient; the test requires minimal human resources and reports results within two hours. The majority of performance studies of MTB/RIF have been performed in high HIV burden settings, thus TB diagnostic studies among HIV patients in low HIV prevalence settings such as Peru are still needed.

**Methodology/Principal Findings:**

From April 2010 to May 2011, HIV-positive patients with high clinical suspicion of TB were enrolled from two tertiary hospitals in Lima, Peru. Detection of TB by MTB/RIF was compared to a composite reference standard Löwenstein-Jensen (LJ) and liquid culture. Detection of rifampicin resistance was compared to the LJ proportion method. We included 131 patients, the median CD4 cell count was 154.5 cells/mm^3^ and 45 (34.4%) had TB. For TB detection among HIV patients, sensitivity of MTB/RIF was 97.8% (95% CI 88.4–99.6) (44/45); specificity was 97.7% (95% CI 91.9–99.4) (84/86); the positive predictive value was 95.7% (95% CI 85.5–98.8) (44/46); and the negative predictive value, 98.8% (95% CI 93.6–99.8) (84/85). MTB/RIF detected 13/14 smear-negative TB cases, outperforming smear microscopy [97.8% (44/45) vs. 68.9% (31/45); p = 0.0002]. For rifampicin resistance detection, sensitivity of MTB/RIF was 100% (95% CI 61.0–100.0) (6/6); specificity was 91.0% (95% CI 76.4–96.9) (30/33); the positive predictive value was 66.7% (95% CI 35.4–87.9) (6/9); and the negative predictive value was 100% (95% CI 88.7 –100.0) (30/30).

**Conclusions/Significance:**

In HIV patients in our population with a high clinical suspicion of TB, MTB/RIF performed well for TB diagnosis and outperformed smear microscopy.

## Introduction

Tuberculosis (TB) is the leading cause of death in human immunodeficiency virus (HIV) infected patients [1]. New TB diagnostic tests and strategies are urgently needed within this population. Several new TB diagnostic tests have recently been developed; however, those require further evaluation among HIV infected patients. Ideally these new diagnostic tests should be accurate, provide results in a time frame that allows efficient treatment decision-making without increasing the demand of the already scarce human resources available in countries affected by HIV and TB.

Achieving accurate diagnosis of TB disease is more complex in HIV patients than in subjects with normal immunity [2]. Sputum smear microscopy has limited accuracy amongst HIV infected patients, further complicated by the multiple clinical, subclinical and atypical presentations observed among these patients [2], [3]. Furthermore, TB disease can disseminate rapidly in patients with advanced immunosuppression. Prompt diagnosis of TB in HIV patients could lead to early treatment initiation and could contribute to decrease TB-related mortality.

Smear microscopy is the cornerstone of TB diagnosis and case detection in the vast majority of TB control programs [4]. It is inexpensive, has few technical requirements and in settings with high burden of disease smear microscopy has a high positive predictive value despite its variable (35 to 80%) sensitivity [5]. Nevertheless, conditions such as high HIV rates, concurrent non-tuberculosis mycobacterial infections, and multidrug resistant tuberculosis (MDR-TB) can impact its diagnostic yield and effectiveness [6], [7]. The current reference standard for TB diagnosis is the culture of *Mycobacterium tuberculosis* (Mtb).

Culture does allow for drug resistance testing; however the test requires proper laboratory infrastructure and trained personnel and the time required for culture growth is long [8].

Xpert®MTB/RIF (Cepheid, Sunnyvale, USA) is a semi-quantitative molecular test for simultaneous detection of TB and rifampicin resistance through detection of the rpo*B* gen. This test works with the GeneXpert® System device (Cepheid, Sunnyvale, USA) that fully automates a real-time polymerase chain reaction (rt-PCR) and provides results within two hours. It has minimal biosafety requirements and reduced technical manipulation [9]. Xpert®MTB/RIF was endorsed in 2010 by the World Health Organization (WHO) for the screening of TB in persons suspected of having MDR-TB or HIV-TB co-infection.

To date, most studies have evaluated the performance of Xpert®MTB/RIF test (from now on referred as MTB/RIF) in pulmonary and extrapulmonary specimens mostly in HIV-endemic countries in Africa where up to 80% of TB patients are HIV co-infected [10]–[17]. We evaluated the performance of MTB/RIF in HIV-positive adult patients with high clinical suspicion of pulmonary TB in two sites in Lima, a setting that has one of the highest TB and MDR-TB rates in the Americas, as well as low (<3%) HIV prevalence in the general population.

## Methods

### Study Setting

We conducted a cross-sectional study to evaluate the diagnostic test accuracy of MTB/RIF in identifying pulmonary TB disease in HIV patients in two tertiary hospitals: Hospital Nacional Hipólito Unánue (HNHU) in Eastern Lima and Instituto de Medicina Tropical Alexander von Humboldt (IMTAvH) in Northern Lima. In 2010, the incidence for all TB cases in Peru was 110 per 100,000 population and 2.6% were co-infected with HIV [18].

### Study Patients

We included patients 18 years of age or older with an HIV diagnosis confirmed by Western Blot, a high clinical suspicion of TB and who had not received more than two doses of TB treatment. A high clinical suspicion of TB was defined as cough for ten or more days with concurrent abnormal chest x-ray (cavity, focal opacity, pleural effusion, nodule or lymphadenopathy) and at least one of the following symptoms: fever, fatigue, night sweats, hemoptysis, chest pain or weight loss. We included those who agreed to participate and completed the written informed consent. Patients who did not provide a second sputum sample with the required volume were subsequently excluded.

### Study Procedures

Trained study health personnel interviewed and enrolled study patients using a structured questionnaire for demographic, clinical and epidemiological data. Interviews were conducted prior to obtaining the first sputum sample. Clinical records were reviewed in case of discrepancies between the reference standard and the MTB/RIF.

### Sample Collection and Processing

The microbiology laboratory at IMTAvH conducted diagnostic tests requested by the Peruvian National TB Program [smear microscopy, Löwenstein-Jensen culture (LJ), and LJ proportion method (LJ PM)] and Mycobacteria Growth Indicator Tube (MGIT). Routine tests done on the first sputum sample, usually an on-the-spot sample, were not included in the primary analysis. The following day, the second sample, usually a morning sample, was collected and used to perform direct MTB/RIF and to repeat all the tests done to the first sample. Sputum samples that could not be processed on the same day were stored at 4°C and processed the following morning or on Monday if it was collected on a Saturday.

The study staff transported all the samples by car on a daily basis at 4°C from HNHU to the microbiology laboratory at IMTAvH (distance of ∼20 minutes). All tests were performed according to standard protocols and established guidelines [19], [20]. Briefly, 3 ml of sputum were transferred to a 50 ml Falcon tube to be decontaminated with N-acetyl-L-cysteine and sodium hydroxide; of the decontaminated pellet ∼0.5 ml was used for smear staining with Ziehl-Neelsen. Two slopes of LJ culture were inoculated with ∼0.2 ml sputum pellets. For MGIT, ∼0.5 ml sputum pellets were inoculated on liquid medium BD BBL Manual MGIT™ (Cockeysville, MD, USA) and MGIT tubes were read using the BD BACTEC™ MicroMGIT Fluorescence Reader (Cockeysville, MD, USA). Drug susceptibility testing was performed using the LJ PM. For direct MTB/RIF, the sputum sample was carefully mixed to make it homogeneous, then sample reagent was added to 1 ml of untreated sputum on a 2∶1 ratio, mixed twice manually during the incubation period for 15 minutes at room temperature, and then 2 ml were transferred to the MTB/RIF cartridge as previously described [21]. The cartridge was closed and placed into the GeneXpert® System for analysis.

Three trained laboratory technicians performed routine tests and the MTB/RIF test was performed by a single technician with experience in handling the GeneXpert® System. All technicians remained blinded to results of the tests they did not perform.

Due to test manufacturer modifications to MTB/RIF software during study performance, we worked with two different MTB/RIF software versions: 2.1 and 4.0. The new version (4.0) had higher cutoff values for rifampicin detection and did not include changes for Mtb detection [16]. The new version was used for only six samples included in this analysis.

### Data Management and Statistical Analysis

A head-to-head per-sample analysis of MTB/RIF was the primary analysis for Mtb detection: the second sputum sample was examined using the MTB/RIF as the index test and the reference standard was a composite culture (LJ/MGIT) of the second sputum sample. The reference standard was positive if there was Mtb growth in at least one slope of LJ or in a MGIT culture tube, and negative if the results of both cultures - LJ and MGIT- were negative. In addition, the reference test was considered contaminated if both LJ and MGIT were contaminated. Contaminated reference standard tests or a MTB/RIF result reported as invalid were excluded from the analysis. We compared the performance of MTB/RIF with that of sputum smear microscopy. Finally, considering the LJ PM as the reference standard, we assessed the performance of MTB/RIF for the detection of rifampicin resistance and MDR-TB. Accordingly, a rifampicin resistance case was defined as rifampicin resistance detected by the LJ PM and rifampicin sensitive case was defined if the results of the LJ PM showed patterns of full drug sensitivity or drug resistance excluding rifampicin.

As a secondary analysis we conducted a head-to-head per-patient assessment for Mtb detection. A subject with a positive reference standard test in at least one of the two sputum samples was considered a PTB case, and one with a negative reference standard test in both sputum samples was not considered a PTB case. Unless we refer to a “PTB case”, all other mentions to culture-positive patients or patients with tuberculosis refers to the primary analysis, thus to the second sputum sample.

Study sample size was calculated using sample size formula for estimating a proportion with a normal approximation n = z^2^.p(1-p)/e^2^ [22] with expectations of 98% sensitivity and 97% specificity (chosen from reports on MTB/RIF in HIV-negative patients [21] as at the time that our study was designed there were no studies on HIV-positive patients), 5% desired precision for a 95% confidence interval, and 5% expected attrition. No power level was specified because the primary objective was estimation and not a comparison.

All data from questionnaires and laboratory results was entered into Microsoft Access database (Microsoft, Redmond, WA, USA). Sensitivity, specificity, likelihood ratios (LLR), kappa coefficient and predictive values of the tests were calculated using 2×2 tables and OpenEpi v 2.3.1 [23] and the Wilson score method was used to obtain 95% confidence intervals (CI). This report was done following STARD guidelines [24].

### Ethical Considerations

The study protocol was approved by the Institutional Ethics Committee at Universidad Peruana Cayetano Heredia and by the Institutional Ethics Committee at HNHU. Written informed consent was received from all participants and all data was processed anonymously. MTB/RIF results were not used for treatment management; only routine tests results were given to the treating physicians. TB cases were then referred to the National TB Program center at each site where free treatment under directly observed treatment short course (DOTS) was provided.

## Results

**Figure 1:**
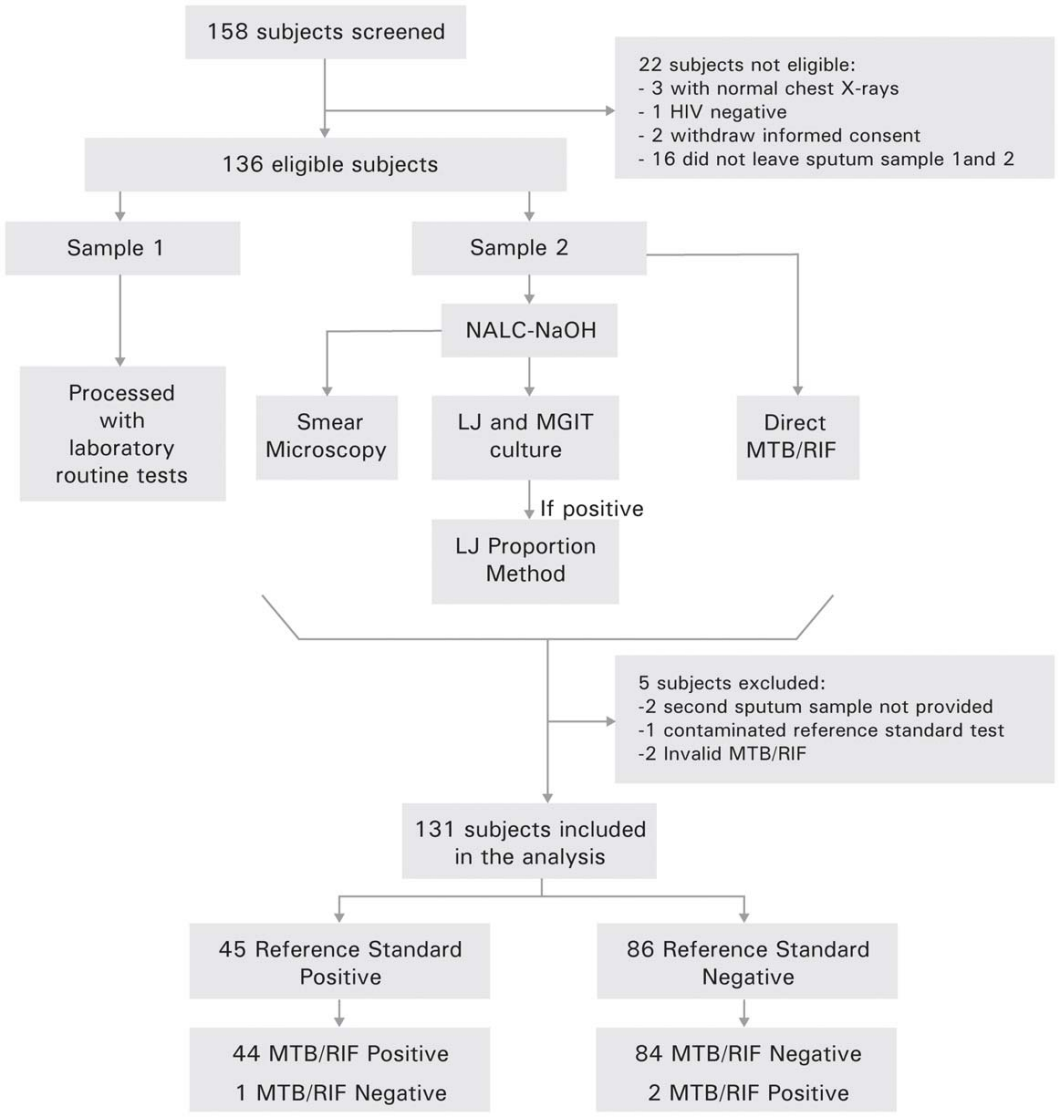
Study Algorithm. LJ: Löwenstein-Jensen culture; MGIT: Mycobacteria Growth Indicator Tube; Reference standard: composite LJ & MGIT culture; MTB/RIF: Xpert®MTB/RIF; Routine tests: Smear, LJ, MGIT and LJ proportion method; NALC-NaOH: N-acetyl-L-cysteine and sodium hydroxide.

From April 2010 to May 2011, 158 patients were screened in the two sites, of which 136 were eligible for the study and 131 were included in the analysis, as shown in figure 1. The median age of patients was 35 years (IQR 29–42) and 73% were male. The median CD4 count was 154.5 cells/mm^3^ (IQR 51.3–341.5). A prior TB episode was reported by 25% of patients and 32% were receiving highly active antiretroviral therapy (HAART) at enrollment. Other demographic data is listed in table 1.

**Table 1:** Selected demographic characteristics of study patients.

	Total Study Patients (N = 131)	Culture Negative(N = 86)	Culture Positive(N = 45)	Crude RR (CI95%)
**Median age in years (IQR)**	35(29–42)	35(30–42)	34(29–41)	
**Median CD4 count** * **(IQR)**	154.5 (51.5–341.5)	124 (37.5–346.0)	222 (87.0–339.0)	
	n/N (%)	n/N (%)	n/N (%)	n/N (%)
**Gender Male Female**	95(73) 36(27)	61(71) 25(29)	34(76) 11(24)	1.1(0.8–1.4) 1
**Prior TB episode Yes No**	33(25) 98(75)	20(23) 66(77)	13(29) 32(71)	1.1(0.8–1.5) 1
**On HAART at enrollment Yes No**	42(32) 89(68)	30(35) 56(65)	12(27) 33(73)	0.9(0.7–1.1) 1
**Household contact Yes No**	36(27) 95(73)	20(23) 66(77)	16(36) 29(64)	1.3(0.9–1.7) 1
**Previously received isoniazid preventive treatment** ŧ **Yes No**	15(12) 112(85)	13(15) 73(85)	2(4) 39(87)	0.4(0.1–1.4) 1

IQR: interquartile range; TB: tuberculosis; HAART: Highly active antiretroviral therapy.

* Excludes five patients with no CD4 count data.

ŧExcludes four patients with unknown information about isoniazid preventive treatment.

Out of the 131 patients included, 45 (34.4%) had TB and among these 14 (31.1%) were smear negative. The proportion of TB per site was 45.1% (95% CI 31.4–58.6) at HNHU and 27.5% (95% CI 18.6 –38.0) at IMTAvH.

### Mtb Detection with MTB/RIF

Overall, MTB/RIF sensitivity for detection of Mtb was 97.8% (95% CI 88.4–99.6) (44/45); the specificity, 97.7% (95% CI 91.9–99.4) (84/86); the positive predictive value, 95.7% (95% CI 85.5–98.8) (44/46); and the negative predictive value, 98.8% (95% CI 93.6–99.8) (84/85). The positive likelihood ratio (+LLR) of MTB/RIF was 42.0 (95% CI 15.8–112.1), and the negative LLR 0.0 (95% CI 0.0–0.2). The kappa coefficient value was 0.9 (95% CI 0.8–1.1).

MTB/RIF outperformed smear microscopy [97.8% (44/45) vs. 68.9% (31/45); p = 0.0002] and detected 13 out of 14 (92.2%) smear negative, culture-positive versus 31 out of 31 (100%) smear-positive, culture-positive patients. Table 2 shows the distribution of combined results according to reference standard, index test and smear microscopy for the 131 patients included in the analysis as well as three additional eligible patients with indeterminate results.

**Table 2:** Combinations of smear microscopy, reference standard and MTB/RIF results within eligible patients.

Number of patients (%)*	Reference standard		Index test(MTB/RIF)	Smearmicroscopy	Comment
	Löwenstein- Jensen	MGIT			
**30 (22.4)**	**+**	**+**	**+**	**+**	full agreement
**13 (9.7)**	**+**	**+**	**+**	**–**	false negative smear
**1 (0.7)**	**+**	**+**	**–**	**–**	false negative MTB/RIF and smear
**1 (0.7)**	**–**	**–**	**+**	**+**	false positive MTB/RIF and smear
**2 (1.5)**	**–**	**–**	**–**	**+**	false positive smear
**1 (0.7)**	**–**	**–**	**+**	**–**	false positive MTB/RIF
**1 (0.7)**	**–**	**+**	**+**	**+**	false negative LJ
**82 (61.2)**	**–**	**–**	**–**	**–**	full agreement
**1 (0.7)**	contaminated	contaminated	**–**	**–**	contaminated reference standard
**2 (1.5)**	**–**	**–**	invalid	**–**	invalid MTB/RIF

Löwenstein-Jensen = LJ; MGIT = Mycobacteria Growth Indicator Tube; MTB/RIF = Xpert®MTB/RIF;

<$>\raster="rg2"<$> = positive result; <$>\raster="rg3"<$> = negative result; LJ and MGIT: composite reference standard.

* Two eligible patients were excluded because they did not provide a second sputum sample.

### Rifampicin Resistance Detection with MTB/RIF

Six MTB/RIF cartridges with improved rifampicin resistance software (v 4.0) were used but they were all Mtb negative for both reference standard and MTB/RIF tests. Rifampicin resistance was assessed in 39 (86.7%) out of 45 patients with TB. In five cases the results of the LJ PM were not available (five LJ PM were not done, due to clerical error and one LJ PM was sensitive for all drugs, but MTB/RIF was negative for Mtb).

Rifampicin resistance was found in six out of 39 patients, all of these were also detected by MTB/RIF: five had MDR-TB and one was sensitive to isoniazid. Furthermore, MTB/RIF detected rifampicin resistance in three additional patients that were not detected by the reference standard. Overall, MTB/RIF sensitivity for rifampicin detection was 100% (95% CI 61.0–100.0) (6/6); the specificity was 91.0% (95% CI 76.4–96.9) (30/33); the positive predictive value was 66.7% (95% CI 35.4–87.9) (6/9); and the negative predictive value 100% (95% CI 88.7 –100.0) (30/30). The +LLR was 11 (95% CI 5.7–21.1), and the kappa coefficient value was 0.8 (95% CI 0.5–1.1).

### Outcomes of Patients with Discordant Results

In terms of Mtb detection by MTB/RIF, three (2.3%) patients had discordant results with the reference standard. A false negative MTB/RIF test was observed in a patient with a negative smear. The patient showed a positive response to treatment (defined as resolution of initial symptoms and weight gain) and was reported as cured. Two patients had false positive MTB/RIF tests, one of them was also smear-positive, responded well to treatment and was reported as cured; the other did not start treatment and died one month after study inclusion with no defined cause of death.

Finally, there were two cases with positive smears and negative results in the reference standard and MTB/RIF tests. Both of them completed TB treatment and were reported as cured.

Three patients were MTB/RIF rifampicin resistant and LJ PM sensitive; all three started TB treatment for sensitive cases (isoniazid, rifampicin, pyrazinamide and ethambutol for two months followed by isoniazid and rifampicin for four months). Two of them finished treatment and were reported to be cured and the other one was lost to follow-up.

### Performance of MTB/RIF by Immunological Status of Patients

Performance of MTB/RIF by immunological status of the patients was evaluated for 96.2% (126/131) patients with available CD4 count at study inclusion. MTB/RIF performance was not affected by immunological status. Patients with CD4 counts below 200 cells/mm^3^ had a sensitivity of 100% (95%CI 83.9–100.0) (20/20) while patients with CD4 counts above 200 cells/mm^3^ had a sensitivity of 95.5% (95%IC 78.2–99.1) (21/22); p = 0.5. Patients with CD4 counts below 200 cells/mm^3^ had a specificity of 96.1% (95%CI 86.8–98.9) (49/51), while patients with CD4 counts above 200 cells/mm^3^ had a specificity of 100% (95%IC 89.6–100.0) (33/33); p = 0.4.

### Indeterminate Results

One patient had a contaminated reference standard test (1/134, 0.7%) with a negative MTB/RIF result. Two patients (2/134, 1.5%) had invalid results with MTB/RIF (which translates into the sample not properly processed or rt-PCR inhibited). As sufficient sample remained, MTB/RIF test was repeated in these two patients, without detection of Mtb in concordance with the results of their reference standard.

### Analysis Including Two Sputum Samples

We assessed performance of the index test for Mtb detection, done only on the second sputum sample, with a reference standard defined as any positive result in either the first or the second sputum sample to have a per-patient analysis.

MTB/RIF sensitivity for detection of Mtb was 86.3% (95% CI 74.3–93.2) (44/51); the specificity, 97.5% (95% CI 91.3–99.3) (78/80); the positive predictive value, 95.7% (95% CI 85.5–98.8) (44/46); and the negative predictive value, 91.8% (95% CI 84.0–96.0) (78/85). The +LLR of MTB/RIF was 34.6 (95% CI 12.9–92.6), and the negative LLR was 0.1 (95% CI 0.1–0.2). The kappa coefficient value was 0.9 (95% CI 0.7–1.0).

A comparison of the performance of MTB/RIF for Mtb detection between per-sample and per-patient analysis is described in Table 3.

**Table 3:** MTB/RIF performance for *Mycobacterium tuberculosis* detection in per-patient and per-sample analysis.

	MTB/RIF per-patient*	MTB/RIF per-sampleŧ
**Sensitivity**	86.3% (95% CI 74.3–93.2) (44/51)	97.8% (95% CI 88.4–99.6) (44/45)
**Specificity**	97.5% (95% CI 91.3–99.3) (78/80)	97.7% (95% CI 91.9–99.4) (84/86)
**Positive predictive value**	95.7% (95% CI 85.5–98.8) (44/46)	95.7% (95% CI 85.5–98.8) (44/46)
**Negative predictive value**	91.8% (95% CI 84.0–96.0) (78/85)	98.8% (95% CI 93.6–99.8) (84/85)

MTB/RIF = Xpert®MTB/RIF.

* The per-patient analysis evaluated the performance of MTB/RIF results from the second.

sample only against results from Löwenstein-Jensen (LJ) and Mycobacteria Growth Indicator.

Tube (MGIT), from both first and second sputum samples.

ŧ The per-sample analysis was done on the second sputum sample and evaluated the performance.

of MTB/RIF against the results from LJ and MGIT from the second sputum sample.

## Discussion

We report that MTB/RIF had a high specificity (97.7%) in detecting Mtb, confirming the findings of other studies [11], [12], [14], [15]. Two patients had false positive MTB/RIF results as compared to the reference standard; however, one of them was clinically diagnosed with TB and successfully completed treatment. In low income countries, TB diagnosis and treatment initiation is based on smear microscopy results. In our study, MTB/RIF outperformed smear microscopy for Mtb detection in almost one third of the patients. One could expect that the prompt results provided by MTB/RIF would allow a timely diagnosis and prompt initiation of TB treatment. As extensively reported in the medical literature, the benefits of rapid treatment initiation of TB in HIV co-infected patients could improve individual prognosis and reduce overall TB disease transmission [2], [25]. Our results indicate much better MTB/RIF performance in Mtb detection than what has been reported by other studies on HIV patients [12]–[15] which may reflect some differences in study or diagnostic methodologies, or that our study population had a higher probability of TB disease, illustrated by the presence of suggestive symptoms including at least 10 day cough and chest-x ray abnormalities and the high number of patients that were not on HAART at the time of enrolment despite their compromised immunological status.

A lower sensitivity (73%) of MTB/RIF was found among patients with or without TB symptoms in an antiretroviral therapy clinic in South Africa [15]. These patients had a lower probability of TB as opposed to our population. In a multicenter study with 40% of co-infected patients, the MTB/RIF test attained a sensitivity of 94%, similar to ours [11].

Three previous studies - two in South Africa and one in Tanzania- reported sensitivities of 70%, 84% and 88% respectively, while in our setting the sensitivity to detect TB was 98% [12]–[14]. These differences could also be partially explained by the fact that these studies were done on frozen stored samples. Prolonged sample storage and freeze thaw cycles may damage Mtb DNA and affect sputum viscosity, although a recent study done on frozen sputum samples described that MTB/RIF detected 64 out of 85 (75.3%) smear negative, culture-positive sputum samples, suggesting that freezing may have little impact on MTB/RIF sensitivity [26]. Nevertheless, this should be confirmed in larger studies.

Despite all the advances of HAART scale up worldwide, much of the preventable burden of TB related mortality is concentrated in populations with advanced immunosuppression and without HAART, as that of this study. Only 32% of HIV patients had initiated HAART at the time of enrolment in our study.

When we analyzed the performance of MTB/RIF compared to a reference standard including results from both sputum samples, the sensitivity was considerably reduced, yet this could be related to the fact that MTB/RIF was only done in one sample.

The performance of MTB/RIF to detect rifampicin resistance and thus its contribution for MDR-TB detection were not equally convincing. The index test did detect all the rifampicin resistant cases but also reported three false positives. Previous studies have addressed this issue [9], [15], [16], [27] and the WHO recommends that rifampicin resistance results of MTB/RIF should be confirmed with further tests and treatment regimens should be based on the latter [28].

Our study has some limitations. WHO new guidelines recommend that TB should be suspected in any HIV-positive individual with any of the following symptoms: cough, weight loss or fever. Our study was designed before these guidelines were set, and it aimed to evaluate performance of MTB/RIF in a group of HIV-positive individuals with at least two of these symptoms, thus a more selected population. We decided to study a selected population of HIV-positive patients to narrow the risk of tuberculosis to a higher one. MTB/RIF performance could decrease among a less selected population of HIV-positive individuals as compared to our results. Currently, MTB/RIF is still costly and targeting its use in patients with the highest risk of TB could be a strategy for resource-limited settings.

Due to resource limitations, we only evaluated MTB/RIF performance on a single sputum sample; we could not genotype the strains of three cases with false positive rifampicin resistance results. However this reflects the commonly available resources in settings with high prevalence of TB. Also, the three false positive tests were performed with MTB/RIF software v 2.1 and not with the improved software v 4.0. Nonetheless, false positive rifampicin results have been previously reported with the latest version [15]. Finally, our sample size was small for a precise assessment of MTB/RIF performance for rifampicin resistance detection. The results we report may be extrapolated to populations similar to ours but not necessarily to others with lower pre-test probability such as HIV patients without specific symptoms suggestive of TB. However these study findings suggest that in a similar setting and context an MTB/RIF negative, HIV-positive patient can be treated with high confidence.

In our study, MTB/RIF showed an excellent performance in detecting TB among patients with advanced immunosuppression and a high clinical suspicion of TB. A positive MTB/RIF result was almost 40 times more likely to occur in a subject with TB than in a subject without TB, and a negative MTB/RIF was also much more commonly seen in patients without TB.

We conclude that MTB/RIF can be an important diagnostic tool for TB disease amongst HIV-positive patients, particularly in patients with a high pre-test probability of TB. Many studies of new rapid TB diagnostic tests have been conducted in Africa where high HIV rates place a different perspective on TB programs and health systems. Further evaluation of MTB/RIF in Latin America is needed. Operational research should evaluate the yield of scaling up diagnostic algorithms of such strategies in order to evaluate the cost-effectiveness of rapid treatment initiation, improvement of individual prognosis and reduced disease transmission, within well established tuberculosis programs in TB endemic settings [29], [30].
